# Novel Cytotoxic Vectors Based on Adeno-Associated Virus

**DOI:** 10.3390/toxins2122754

**Published:** 2010-12-01

**Authors:** Johannes Kohlschütter, Stefan Michelfelder, Martin Trepel

**Affiliations:** Hubertus Wald Cancer Center, Department of Oncology and Hematology, University Medical Center Hamburg-Eppendorf, Martinistr. 52, 20246 Hamburg, Germany; Email: johannes.kohlschuetter@online.de (J.K.); s.michelfelder@uke.de (S.M.)

**Keywords:** adeno-associated virus, cytotoxic gene therapy, vector targeting

## Abstract

Vectors based on adeno-associated virus (AAV) are promising tools for gene therapy. The production of strongly toxic vectors, for example for cancer-directed gene transfer, is often unfeasible due to uncontrolled expression of toxic genes in vector-producing cells. Using an approach based on transcriptional repression, we have created novel AAV vectors carrying the genes coding for diphtheria toxin A (DTA) and the pro-apoptotic PUMA protein. The DTA vector had a significant toxic effect on a panel of tumor cell lines, and abrogation of protein synthesis could be shown. The PUMA vector had a toxic effect on HeLa and RPMI 8226 cells, and sensitized transduced cells to doxorubicin. To permit targeted gene transfer, we incorporated the DTA gene into a genetically modified AAV-2 capsid previously developed by our group that mediates enhanced transduction of murine breast cancer cells *in vitro*. This vector had a stronger cytotoxic effect on breast cancer cells than DTA vectors with wildtype AAV capsid or vectors with a random capsid modification. The vector production and application system presented here allows for easy exchange of promotors, transgenes and capsid specificity for certain target cells. It will therefore be of great possible value in a broad range of applications in cytotoxic gene therapy and significantly broadens the spectrum of available tools for AAV-based gene therapy.

## 1. Introduction

Vectors derived from adeno-associated virus (AAV) hold promise as safe and efficient tools for gene therapy. Their desirable safety profile has been confirmed in a number of clinical trials [1]. Vector administration does not elicit a strong cellular immune reaction [2], and the risk of unintended integration into the human genome is low [3,4]. Research efforts aimed at clinical application of AAV vectors are focused on increasing target specificity and transduction efficiency on the one hand and on evaluating suitable therapeutic transgenes on the other. The spectrum of strategies in the former field is wide [5,6]. It includes the exploitation of the natural diversity of AAV serotypes [7], the insertion of targeted peptides [8,9,10], the use of bispecific conjugates [11,12,13], and the insertion of random peptide libraries exposed at the capsid surface, which enables the identification of suitable capsid variants by biopanning [14,15,16,17,18].

The development of AAV vectors for cancer therapy has hitherto been focused on anti-angiogenic, immunomodulatory, and suicide genes, or on genes that mediate cell repair [19]. Cell death has been achieved using the herpes simplex virus thymidine kinase (HSV-tk) gene [20,21,22]; which exerts its toxic effect only after ganciclovir administration, as well as the pro-apoptotic TRAIL gene [23,24].

We set out to expand the arsenal of toxic AAV vectors. The production of vectors carrying strong toxic genes can be hampered by uncontrolled expression of these genes in the cell lines used for packaging. While specific promoters may be an option to circumvent this problem, they may not be available for a given target cell type of interest or may not be strong enough to achieve adequate transgene expression levels in the target cell [25]. We thus aimed to establish a system for the production of cytotoxic AAV vectors under the control of a strong unspecific promoter. Our approach is based on transcriptional repression of toxic genes by a Tet repressor/operator system [26]. We used this system to produce novel cytotoxic vectors containing the genes coding for the catalytically active fragment of diphtheria toxin (DTA) [27] and the pro-apoptotic PUMA protein [28]. 

## 2. Materials and Methods

### 2.1. Cell Lines

Cells were cultured in a humidified atmosphere at 37 °C and 5% CO_2_. HEK293T cells (kindly provided by David Baltimore, California Institute of Technology, Pasadena, CA), HeLa cells (obtained from American Type Culture Collection (ATCC), Manassas, VA), and SiHa cells (kindly provided by Jens Hasskarl, University of Freiburg Medical Center, Freiburg) were cultured in Dulbecco’s Modified Eagle Medium (Invitrogen, Carlsbad, CA) containing 10% fetal bovine serum (Biochrom, Berlin, Germany) and 1% of a penicillin/streptomycin solution (100 units/mL penicillin G/100 μg/mL streptomycin, Invitrogen). HEK293T T-REx cells (Invitrogen) were cultured in Dulbecco’s Modified Eagle Medium containing 10% tetracycline-free fetal bovine serum (PAA Laboratories, Pasching), 1% penicillin/streptomycin, and 5 μg/mL blasticidin (Invitrogen). RPMI 8226 cells (obtained from ATCC) were cultured in Iscove’s Modified Dulbecco’s Medium (Invitrogen) containing 10% fetal bovine serum and 1% penicillin/streptomycin.

### 2.2. Primary Murine Breast Cancer Cells

These cells were obtained from 2-3-month-old female PymT mice (Jackson Laboratories, Bar Harbor, MN) [29]. All procedures involving the use and care of animals were performed according to the Guide for the Care and Use of Laboratory Animals published by the US National Institutes of Health (NIH Publication No. 85-23, revised 1996) and the German animal protection code. Primary tumor cells were obtained by mechanical and enzymatic dissociation of solid PymT-induced carcinomas as previously described [17].

### 2.3. pAAVTetO2 Plasmid Backbone

To create pAAVTetO2 (Figure 1), pAAV-MCS (Stratagene, La Jolla, CA) was digested with *Not*I, and the 2891 bp backbone fragment providing the AAV inverted terminal repeat sequences as well as the ampicillin resistance gene was blunted with the *E. coli* DNA polymerase I Klenow fragment and Antarctic phosphatase. This fragment was ligated with the 1133 bp fragment obtained by digesting pcDNA4/TO (Invitrogen) with *Pvu*II and *Nru*I, which provides the CMV promoter, two tetO2 operator sequences, a multiple cloning site (MCS), and a BGH polyadenylation signal.

**Figure 1:**
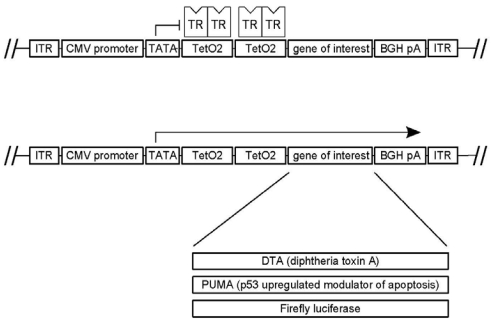
Key features of the pAAVTetO2 plasmid backbone and derivative constructs. The construct provides inverted terminal repeat sequences (ITR), the cytomegalovirus immediate early promoter (CMV), two tet operator sequences (TetO2), and the bovine growth hormone polyadenylation signal (BGH pA). In the presence of the Tet repressor (TR), the transcription of toxic genes is inhibited (top), but it is enabled in its absence (center). The toxic genes and reporter genes cloned into pAAVTetO2 are listed at the bottom.

### 2.4. pAAVTetO2-Based Transgene Constructs

The pAAVTetO2-luciferase construct was obtained by insertion of the 2314 bp fragment containing the firefly luciferase cDNA and an SV polyadenylation signal obtained from the *Xho*I digestion of pTRUFluc [30] into the pAAVTetO2 MCS. To obtain pAAVTetO2-DTA, the gene encoding the catalytic domain of diphtheria toxin A was amplified from pDT201 (ATCC) by PCR, using the forward primer 5'-TCGAAGCTTATGGGCGCTGATGATGTT-3' introducing an ATG start codon and the reverse primer 5'-TCGCTCGAGTCATCGCCTGACACGATT-3' introducing a TGA stop codon [31]. The PCR product was digested with *Hind*III/*Xho*I and inserted into the pAAVTetO2 MCS. To create pAAVTetO2-PUMA, the sequence coding for the murine PUMA protein, with an amino-terminal FLAG tag, was amplified from pEF-PUMA (kindly provided by Christoph Borner, University of Freiburg Medical Center) by PCR using the forward primer 5'-ATGGACTACAAGGACGATGACGACA-3' and the reverse primer 5'-TCGAAGCTTATGGACTACAAGGACGATGACGACA-3'. The PCR product was digested with *Hind*III/*Xho*I and inserted into the pAAVTetO2 MCS. The integrity of toxic gene inserts and flanking regions were verified by sequencing (GATC Biotech, Konstanz).

### 2.5. Production of Recombinant rAAV Vectors

Recombinant AAV vectors were produced in the absence of adenovirus, with *rep* and *cap* genes provided on the pXX2 plasmid and minimal adenoviral helper sequences provided on the pXX6 plasmid [32]. Packaging cells (HEK293T T-REx for toxic constructs, or HEK293T cells for non-toxic reporter constructs) were grown to about 50% confluency and transfected with pXX2, pXX6, and a plasmid carrying the transgene of interest using the PolyFect transfection reagent (Qiagen). For the production of targeted capsid variants, appropriate derivatives of the pXX2-187 plasmid [16] containing a cloning site for the insertion of peptide coding regions at amino acid position R588 were used. 

The transgenes of interest were contained in the pAAVTetO2 constructs created as described above. Alternatively, the pTRUF-CMV-eGFP [15] was used as a reporter construct. Vectors were harvested 3–5 days after transfection by resuspending cells detached from the culture dish in PBS-MK (140 mM NaCl, 5.5 mM KCl, 8 mM Na_2_HPO4, 1.5 mM KH_2_PO4, 1 mM MgCl_2_), followed by cell lysis via three freeze-thaw cycles. Cellular DNA and RNA were removed by incubation with benzonase (Sigma) at >350 U/mL at 37 °C for 30 minutes. Samples were centrifuged, and supernatants containing AAV vectors were stored at −80 °C. If required, vector preparations were purified using the iodixanol gradient centrifugation method [33]. Solutions containing vectors were underlaid with increasing concentrations of iodixanol (15%–54%) and centrifuged at 350,000 × g for 70 minutes, and the 40% iodixanol fraction containing the purified AAV vectors was stored at −80 °C.

### 2.6. Titration of AAV Libraries and Recombinant AAV Vectors

The AAV vector capsid titers were determined as described by an ELISA (Progen, Heidelberg). The genomic titers of recombinant AAV vectors were determined by quantitative PCR using the Absolute SYBR Green fluorescein master mix (Abgene, Epsom) on a MyiQ cycler (Bio-Rad, Hercules, CA), using the primers 5'-GGCGGAGTTGTTACGACAT-3' and 5'-GGGACTTTCCTACTTGGCA-3' specific for the CMV promoter region. The genomic titers of AAV libraries were determined by quantitative PCR, using the primers 5'-GCAGTATGGTGTATCTACCAA-3' and 5'-GCCTGGAAGAACGCCTTGTGT-3' specific for the insert region.

### 2.7. Luciferase Gene Transduction

To analyze luciferase gene transduction, 10^4^ primary PymT tumor cells per well were seeded in 24-well plates and incubated with AAV-luciferase vectors at an multiplicity of infection (MOI) of 10^4^ vector genomes/cell. 72 hours later, luciferase activities in cellular lysates were measured in a luminometer (Berthold Centro LB 960) using the Promega luciferase assay (Promega, Mannheim) according to the manufacturer’s instructions. Values were normalized to protein levels in each probe determined by a Bradford assay (Bio-Rad). For analysis of pAAVTetO2-luciferase constructs, 293T T-REx cells were seeded at a density of 5 × 10^3^ cells per well and were transfected with 80 ng of DNA per well using Lipofectamine (Invitrogen). One day after transfection, the medium was replaced by fresh medium with or without 1 µg/mL tetracycline. Luciferase activity in one-tenth of the lysate volume obtained from each well was analyzed.

### 2.8. Flow Cytometry

To analyze gene transduction by AAV vectors harboring the enhanced green fluorescent protein (GFP), 5 × 10^4^ cells per well of PymT cells were seeded in 24-well plates and incubated with AAV-GFP vectors at an MOI of 10^3^ or 10^4^ vector genomes/cell. After three days, cells were harvested and GFP reporter gene expression was determined using a flow cytometer (FACSCalibur, BD Biosciences, Heidelberg) and CellQuest Pro analysis software. For detection of apoptotic and necrotic processes, RPMI 8226 cells were stained with an annexin V-GFP conjugate and with propidium iodide. GFP-/PI- cells were gated as viable, GFP+/PI- cells were gated as apoptotic, and GFP+/PI+ cells were gated as necrotic. For analysis of the inhibition of GFP synthesis, 2 × 10^4^ cells were seeded.

### 2.9. Viability/toxicity Assay

Toxic effects on cells were analyzed using a colorimetric assay based on the reduction of MTT (3-(4,5-Dimethylthiazol-2-yl)-2,5-diphenyltetrazolium bromide) to a formazan salt in viable cells [34,35]. Cells were incubated with medium containing 500 µg/mL MTT (Invitrogen) for four hours, and the absorbance of formazan crystals dissolved in SDS/HCl was measured at 570 nm in a spectrophotometer.

### 2.10. Statistics

The significance of results was analyzed using Student’s *t*-test (one-sided) at α = 5%.

## 3. Results

### 3.1. A Novel Tet-Regulated Plasmid for Production of Toxic AAV Vectors

We created a novel plasmid construct for production of AAV vectors carrying toxic genes under the control of the cytomegalovirus immediate early promoter. This construct, designated pAAVTetO2, provides the inverted terminal repeat sequences required for packaging of transgenes into vector capsids, as well as tet operator sequences to allow for transcriptional inhibition of transgenes by the Tet repressor (Figure 1). The repressor can be inactivated by addition of tetracycline. To verify that transcription from pAAVTetO2 constructs can be inhibited by the Tet repressor/tet operator mechanism, we transfected HEK293T T-REx cells stably expressing the Tet repressor with a pAAVTetO2 construct containing the firefly luciferase reporter gene (pAAVTetO2-luc), and luminescence was assayed. In the absence of tetracycline, luciferase activity amounted to less than 2% of the value observed in the presence of tetracycline (see Appendix Figure 1), demonstrating that the Tet repressor inhibits transcription efficiently.

### 3.2. Production of Toxic Vectors

**Figure 2:**
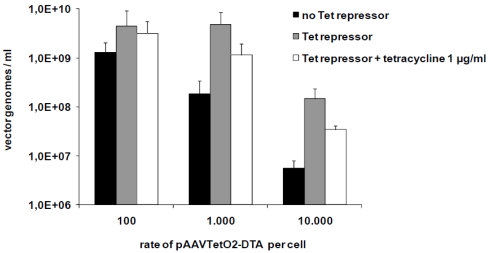
Contributions of the Tet repressor/operator system to increasing titers of toxic vectors. Comparison of titers of vectors carrying the diphtheria toxin A gene (AAVTetO2-DTA) produced under different conditions: without transcriptional repression (black), in the presence of the Tet repressor, and in the presence of the Tet repressor and of 1 µg/mL tetracycline. For each of these conditions, different copy numbers of the DTA gene per packaging cell were evaluated. The data are mean values ± SD from two independent experiments.

Genes coding for DTA and PUMA were cloned into pAAVTetO2. The resulting plasmid constructs were used for the production of toxic vectors with wildtype AAV-2 capsid. Vectors were produced in 293T T-REx cells stably expressing the Tet repressor. Vectors carrying the PUMA gene (AAVTetO2-PUMA) could be produced in the same concentrations as non-toxic vectors produced using an identical setting (with titers in the range of 10^11^–10^12^ vector genomes/mL) if the packaging cells (HEK293T) were transfected with plasmid constructs containing the transgene of interest in the range of 5 × 10^4^ plasmids per cell. The titers obtained for DTA vectors were typically two to three orders of magnitude lower, most probably because of residual expression of the DTA protein, which inhibits protein synthesis even in minute quantities. However, titers could be slightly improved by lowering the copy number of the toxic pAAVTetO2-DTA construct in the packaging cell line, with maximum titers achieved for 100–1,000 copies of pAAVTetO2 per cell. We could further show that the presence of the Tet repressor (pcDNA6/TR) actually results in increased titers for DTA vectors, confirming the validity of our approach (Figure 2).

### 3.3. Evaluation of Novel Toxic Vectors

**Figure 3:**
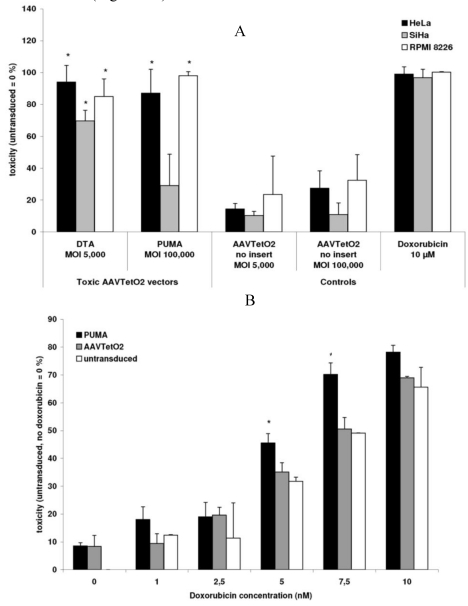
Functionality of novel toxic AAV vectors carrying toxic genes. (A) Toxic effects on a panel of tumor cell lines. The data are mean values ± SD from three independent experiments each done in triplicates; (B) Combined application of the AAVTetO2-PUMA vector and doxorubicin. The data are mean values ± SD from two independent experiments, each done in triplicate. Asterisks indicate a significant toxic effect compared to administration of a non-toxic vector (AAVTetO2).

We evaluated the toxic AAVTetO2-DTA and AAVTetO2-PUMA vectors on HeLa and SiHa cervical carcinoma cell lines, and on the RPMI 8226 myeloma cell line. AAVTetO2-DTA vectors were purified via iodixanol density gradient ultracentrifugation and were evaluated at a maximum multiplicity of infection (MOI) of 5 × 10^3^ vector genomes per cell. The AAVTetO2-PUMA vector was evaluated at a maximum MOI of 10^5^ vector genomes per cell without further purification. For the analysis of toxic effects, cells were seeded in a 96-well plate at a density of 5 × 10^3^ cells per well one day before transduction. Toxic effects were analyzed by an MTT viability assay five days after transduction. For the AAVTetO2-DTA vector, a significant toxic effect could be detected on HeLa cells, SiHa cells, and RPMI 8226 cells at an MOI of 5 × 10^3^ vector genomes per cell. The AAVTetO2-PUMA vector had a significant effect on HeLa cells and RPMI 8226 cells at an MOI of 10^5^, but not on SiHa cells (Figure 3A).

Considering that the PUMA protein can sensitize cells to additional toxic stimuli [36,37], we evaluated the effects of the AAVTetO2-PUMA vector in combination with doxorubicin administration. For that purpose, RPMI 8226 cells were transduced with AAVTetO2-PUMA at an MOI of 10^4^ vector genomes per cell. Doxorubicin was added to the samples one day after transduction. Five days after transduction, toxic effects were analyzed by an MTT assay. Cells transduced with the AAVTetO2-PUMA vector were more sensitive to doxorubicin at a concentration of 5–10 nM than controls transduced with a non-toxic vector (Figure 3B).

**Figure 4:**
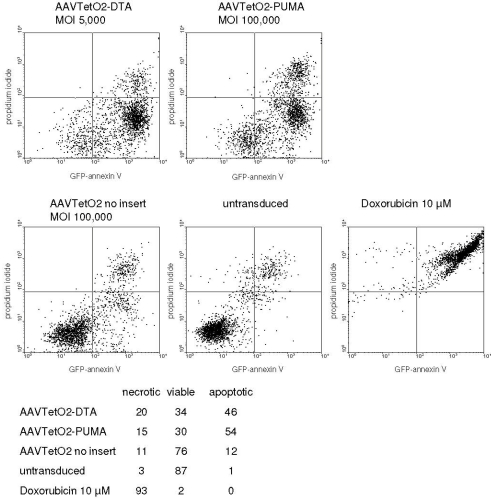
Cell death in cells transduced with DTA and PUMA vectors. Identification of apoptotic, necrotic, and viable cells by flow cytometry. Top: Two-dimensional staining intensity plots. Bottom: Percentage of cells gated as viable, apoptotic, and necrotic (mean values from an experiment done in triplicates).

RPMI 8226 cells transduced with the AAVTetO2-DTA (MOI of 5 × 10^3^ vg/cell) and AAVTetO2-PUMA (MOI of 10^5^ vg/cell) vectors were analyzed by flow cytometry to detect apoptotic and necrotic processes. Five days after transduction, a substantial fraction of cells transduced with toxic vectors, but not with controls, was gated as apoptotic, suggesting that these vectors act via pathways that involve apoptosis (Figure 4).

### 3.4. Targeted Delivery of the DTA Vector to PymT Cells

Once the functionality of the DTA gene when delivered via a wildtype AAV-2 capsid was confirmed, we set out to produce a targeted variant of the DTA vector. For this purpose, we selected a capsid modification previously developed by our group [17] which mediates enhanced *in vitro* transduction of mammary tumor cells from mice carrying the polyomavirus middle T antigen (PymT cells). In this capsid variant, which was identified by biopanning of AAV capsids containing random peptide libraries [17], the amino acid sequence RGDLGLS is inserted at position R588 of the viral capsid protein VP1. The cell-type specificity of RGDLGLS capsids was tested on a panel of non-malignant cell lines and primary murine cells. While 3T3 mouse fibroblasts and primary mouse hepatocytes were not permissive for transduction with RGDLGLS AAV luciferase vectors, they could be efficiently transduced by vectors with wildtype AAV capsids. Further, immunostaining showed that selected AAV vectors selectively transduce cytokeratin positive primary PymT breast cancer cells in primary co-cultured tumor tissue, suggesting target specificity of AAV with RGDLGLS capsid inserts (data not shown).

**Figure 5:**
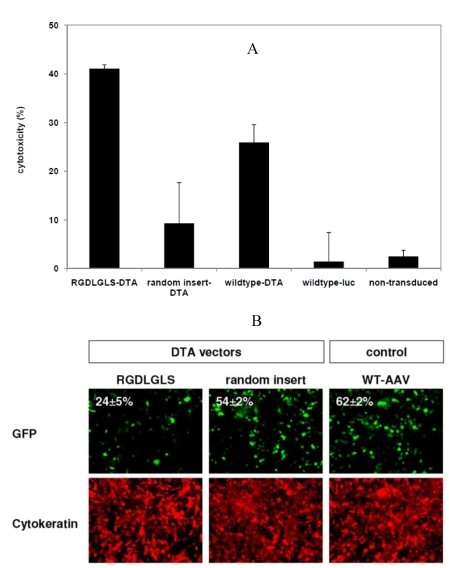
Effects of the diphtheria toxin A gene on murine PymT breast tumor cells, delivered via an AAV vector with targeted capsid structure (RGDLGLS). (A) The targeted vector (RGDLGLS-DTA) has a strong toxic effect on PymT cancer cells compared to a vector with a random sequence capsid insertion or the DTA vectors with wildtype AAV capsid. The data are mean values ± SD from triplicates. (B) Inhibition of eGFP synthesis in PymT cells transduced with the RGDLGLS-DTA vector as assayed by fluorescence microscopy. Cells are visualized by cytokeratine staining. The percentage of cells gated as GFP-positive by flow cytometry in a parallel experiment is indicated. The data are mean values ± SD from triplicates.

We produced vectors with the capsid peptide insertion RGDLGLS and harboring the DTA gene. Vectors with capsids containing the random amino acid sequence VRRPRFW (at position R588) as well as vectors with wildtype capsids were produced as non-targeted controls. PymT primary breast cancer cells were transduced with these vectors at an MOI of 5 × 10^3^. A strong toxic effect of the targeted DTA vectors was detected after four days in an MTT viability assay compared to the effect caused by the control vectors (Figure 5A).

To complement these experiments, we analyzed the inhibitory effect of the targeted vector on protein synthesis. PymT cells were transduced with the RGDLGLS-DTA vector or the control DTA vector, followed by transduction with an RGDLGLS vector harboring the e*gfp* gene. Following DTA transduction, GFP expression was reduced in samples transduced with the RGDLGLS-DTA vector, but not with the control DTA vector, as analyzed by fluorescence microscopy and flow cytometry (Figure 5B).

## 4. Discussion

Production of cytotoxic vectors for therapeutic gene delivery is challenging as uncontrolled cytotoxic gene expression can suppress protein synthesis and/or viability in producer cells. Here, we set out to expand the potential of AAV vectors for experimental tumor therapy. We established and used a transcriptional repression system to produce novel AAV vectors carrying the genes coding for diphtheria toxin A (DTA) and the pro-apoptotic PUMA protein. In several proof of principle experiments, we showed that the vectors produced with our method and carrying the genes coding for the DTA protein and the PUMA protein under the control of the CMV promoter are functional, and both in a targeted and non-targeted vector capsid context.

The characteristics of the vectors presented here differ from those reported for experimental vectors already available. Among the approaches described previously, toxic vectors based on the herpes simplex virus thymidine kinase gene play a prominent role. These vectors exert their effect only indirectly by causing the conversion of a prodrug to a toxic product. In contrast, we aimed to generate toxic vectors that are directly toxic to the cells they transduce, without the need for prodrug administration. Furthermore, we intended to develop vectors with the strongest possible toxic potential that should be applicable to a broad range of cancer cells. Therefore we chose a system delivering toxic genes under the control of a strong and non-tissue-specific promoter such as the CMV promoter, while tissue specificity is supposed to be conferred by a target cell-specific capsid.

To face the challenge of cytotoxicity in vector-producing cells, we propose a novel strategy for production of toxic AAV vectors based on transcriptional repression of toxic genes. While we demonstrate that the system is functional, we recognize that its contribution is limited in the context of genes coding for proteins as potent as diphtheria toxin A, which can be considerably toxic even in minute quantities [40]. Nevertheless, the option to produce vectors carrying toxic genes under the control of a strong and unspecific promoter may be interesting for the evaluation of various toxic candidate vectors on a variety of target cell types. While of broad utility, the system presented here may be further optimized in several ways, depending upon the context in which the vectors are intended for application. In certain target cell types, the system may be further optimized by the use of a specific promoter, if available. In addition, the activity of toxic proteins in the cells used for vector production might be inhibited by transgene-specific means, e.g., via anti-sense/siRNA or via inhibition of the protein A complex. Another approach to further enhance vector productivity may be the generation of toxic vectors in which toxic genes become active only through the excision of spacer DNA by Cre-recombinase delivered via a second vector. This strategy, which has been successfully evaluated using adenovirus [31], may also hold promise in the AAV context.

Broadening the spectrum of toxic vectors is desirable as it provides the option to choose the most efficient mechanism of cell killing depending on the target cells. For instance, the above-mentioned HSV-*tk*-based vectors have been reported to cause toxic effects on untransduced cells in the vicinity of cells expressing HSV-*tk* [38]. This “bystander effect” can be exploited to kill a tumor even if only a fraction of cells can be transduced and even extends to cells of different origin than the initial target cell [22]. In contrast, toxic effects of the DTA gene are restricted to cells that actually express it [39]. This may encourage the further development of DTA-based vectors such as the ones presented here for specific killing of widely disseminated malignant cells, as found in metastatic disease, provided, of course, that target specificity is sufficiently high.

Our data suggest that the toxic vectors described here may be further developed for targeted therapy. While transgene expression in cells other than the actual target may be tolerated in other fields, specificity is paramount in cancer therapy. AAV capsid modifications can improve the transduction efficiency for a target cell type of interest, but in some instances this may lead to increased transduction of other tissues as well [17]. Therefore, our novel cytotoxic AAV vectors are currently being further developed for systemic gene transfer *in vivo* as future advancement in AAV-based experimental cancer therapy will rely on the combination of suitable transgenes with efficient and specific promoters, as well as on the use of receptor-targeted vector capsids.

## 5. Conclusions

Vectors based on adeno-associated virus (AAV) are promising for cancer-directed cytotoxic gene transfer. We produced novel AAV vectors carrying the genes coding for diphtheria toxin A and the pro-apoptotic PUMA protein and demonstrated that these novel vectors can be used to efficiently kill a broad range of tumor cells, both in receptor-targeted and native capsid contexts. Taken together, the vector production and application system presented here broaden the spectrum of tools available for cytotoxic cancer gene therapy based on AAV vectors. The cloning and production system provides excellent versatility in exchanging promotors, transgenes and capsid specificity for certain target cells and will therefore be of great possible value in a broad range of applications in cytotoxic gene therapy.

## Figures and Tables

**Figure 1:**
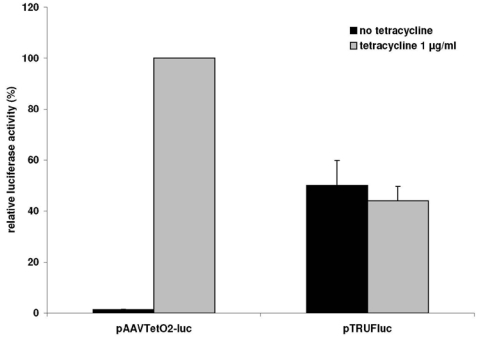
Functionality of transcriptional regulation by the Tet repressor/Tet operator system in the context of the novel construct pAAVTetO2. Analysis of luciferase activity in 293T T-REx cells stably expressing the Tet repressor after transfection with the firefly luciferase gene under the control of the tet operator (pAAVTetO2-luc). Luciferase activity in cells transfected with a construct without tet operator control (pTRUFluc) is shown for comparison. The data are mean values ± SD from two independent experiments, each done in triplicates.
